# Microwave-Assisted One-Step Synthesis of Fenamic Acid Hydrazides from the Corresponding Acids

**DOI:** 10.3390/molecules16053544

**Published:** 2011-04-28

**Authors:** Tarek Aboul-Fadl, Hatem A. Abdel-Aziz, Adnan Kadi, Ahmed Bari, Pervez Ahmad, Tilal Al-Samani, Seik Weng Ng

**Affiliations:** 1 Department of Pharmaceutical Chemistry, College of Pharmacy, King Saud University, P.O. Box 2457, Riyadh 11451, Saudi Arabia; 2 Department of Chemistry, University of Malaya, 50603 Kuala Lumpur, Malaysia

**Keywords:** fenamic acids, fenamic acid hydrazides, microwave irradiation, solvent-free reaction

## Abstract

A facile and efficient method for synthesis of fenamic acid hydrazides from their acids in one-step reaction under microwave irradiation and solvent-free conditions was developed. Compared with the two-step conventional heating method, the process was simple, the reaction time was very short and the yields were almost quantitative.

## 1. Introduction

Flufenamic acid (FFA), *N*-(α,α,α-trifluoro-*m*-tolyl)anthranilic acid, mefenamic acid (MFA), *N*-(2,3-xylyl)anthranilic acid and meclofenamic acid (MCFA), *N*-(2,6-dichloro-3-methylphenyl) anthranilic acid are derivatives of *N*-phenylanthranilic acid (fenamates). They are non-steroidal anti-inflammatory drugs (NSAIDs) used as potent analgesic and anti-inflammatory agents in the treatment of osteoarthritis, rheumatoid arthritis and other painful musculosketal illnesses [1,2,3,4,5]. The fenamates exhibit pharmacologic actions similar to those of aspirin. They are potent inhibitors of cyclooxygenase, thereby inhibiting the release of prostaglandins [5]. Furthermore, *in vitro* and *in vivo* antimycobacterial activities of some NSAIDs such as diclofenac and its derivatives have been also reported [6]. Diclofenac is structurally related to fenamic acids suggesting that these NSAIDs may inhibit a new target in *M. tuberculosis*. On the other hand, hydrazides are important key intermediates in the synthesis of many series of biologically active heterocycles, and their synthesis has attracted significant attention due to their utility as building blocks [7,8,9,10,11] and aroused our interest in exploring the utility of hydrazides as versatile precursors for the synthesis of a variety of substituted heterocycles [12,13,14,15,16,17,18,19,20].

In view of the above facts and as a part of our ongoing project directed to develop new isatin hydrazone anti-TB agents using combinatorial chemistry and microwave-assisted synthesis technologies [21], an efficient, fast and high yielding method for preparing hydrazide building blocks for the design of combinatorial libraries is urgently needed. The current work describes a direct, microwave assisted, one-pot synthesis of some fenamic acid hydrazides from their corresponding fenamic acids. 

## 2. Results and Discussion

**Scheme 1:**
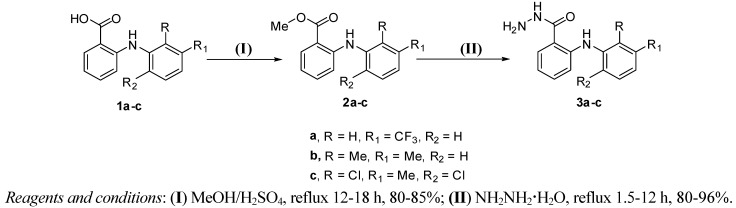
Conventional synthesis of fenamic hydrazides **3a-c**.

Over the last few years, there has been growing interest in the synthesis of organic compounds using green chemistry tools such as microwave irradiation because of increasing environmental consciousness. The feasibility of microwave assisted synthesis has been demonstrated in various transformations whose main features are enhanced reaction rates, greater selectivity and experimental ease of manipulation leading to efficient, environmentally friendly in addition to cost effective synthetic pathways to several compounds [22,23]. Moreover, the use of microwave irradiation in this regard is now a well-established procedure in MORE (microwave induced organic reaction enhancement) chemistry [24]. In the present study, a direct microwave-assisted one-step synthesis of some fenamic acid hydrazides from their corresponding acids was developed. The obtained results were compared with the conventional two-step method. In the latter, esters **2a-c** are obtained in 80-85% yield by esterification of the corresponding fenamic acids, **1a-c**, with methanol, in the presence of sulfuric acid, under reflux for 12-18 h [25,26,27,29]. The second step involves production in 80-96% yield of hydrazides **3a-c** by the reaction of methyl esters **2a-c** with hydrazine hydrate under reflux for 1.5-12 h. However, the combined reaction times of the latter two steps were 15-28 h, with overall yields being 64-86% [27,28,29] (Scheme 1, Table 1). It is worth mention that direct reaction of the fenamic acids, **1a-c**, with hydrazine hydrate to synthesize the hydrazides **3a-c** with the conventional heating method was unsuccessful.

**Table 1:** Reaction times and yield of conventional (2 steps) and microwave-assisted synthesis.

Comp.	Conventional synthesis		MW-assisted synthesis	
	Time of 2 step route (h)	Overall yield (%)	Time (min)	Yield (%)
**3a**	15	80	4	96
**3b**	28	64	12	82
**3c**	17	86	5	85

**Scheme 2:**
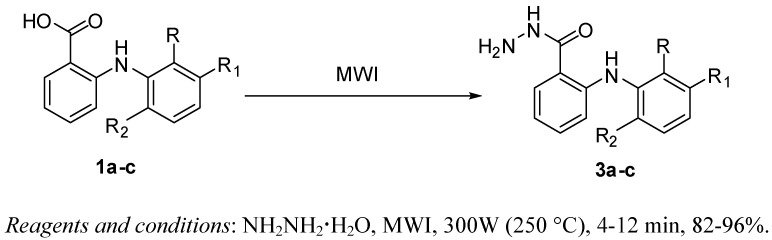
Microwave-assisted one-step synthesis of fenamic hydrazides **3a-c**.

Interestingly, compounds **3a-c** were obtained directly in excellent yields from the reaction of acids **1a-c** with hydrazine hydrate in absence of organic solvents under microwave irradiation (300W, 250 °C) for 4-12 min (Scheme 2, Table 1). Spectral analyses of the synthesized hydrazides **3a-c** are consistent with the proposed structures and with those reported [27,28,29].

**Figure 1:**
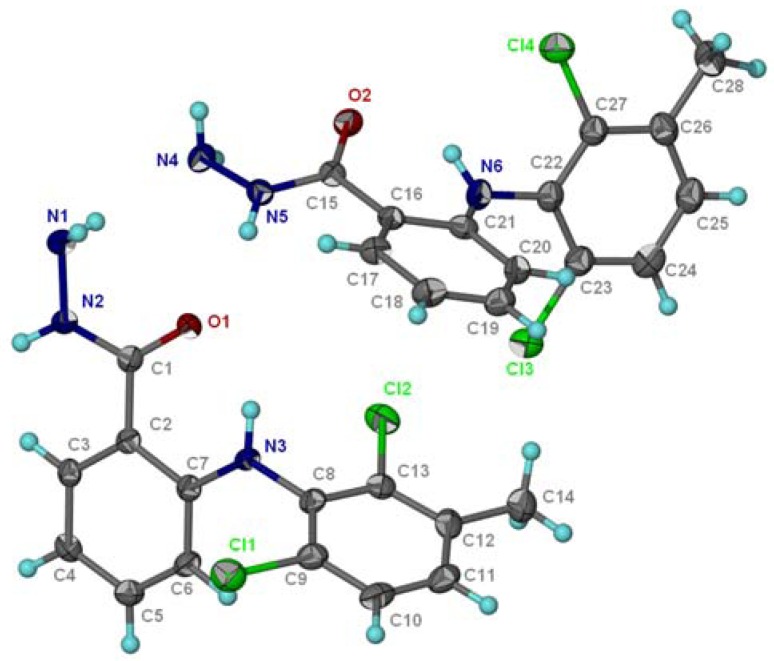
X-ray structure of hydrazide **3c**.

**Figure 2:**
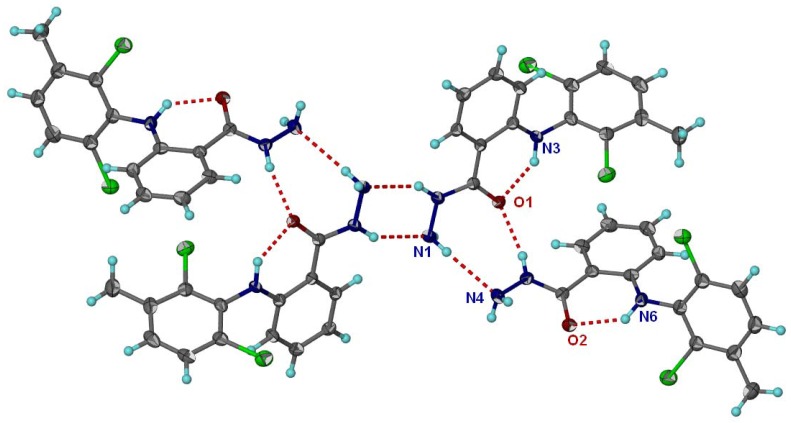
Crystal packing of hydrazide **3c**.

X-Ray diffraction of hydrazide **3c** (Figure 1) [30] showed a network of hydrogen bonds. Two molecules of the dimmer are linked by intermolecular hydrogen bonds (N5-H5∙∙∙∙∙O1 and N1-H1∙∙∙∙∙N4). Besides the latter intermolecular H-bonds, there are two intramolecular H-bonds, one in each molecule of the dimmer, N3–H3∙∙∙∙∙O1 and N6–H6∙∙∙∙∙O2 (Figure 2).

## 3. Conclusions

A microwave-assisted one-step synthesis of some fenamic acid hydrazides by reaction of their corresponding acids with hydrazine hydrate under solvent-free conditions was developed and described. In this method, the reaction times were very short (4-12 min), the yields were excellent (82-96%) and the manipulations were simple compared to the conventional heating method. 

## 4. Experimental

### 4.1. General

Fenamic acids were purchased from the Sigma-Aldrich Company (St. Louis, MO, USA). All other chemicals are of commercially available research-grade. Melting points were determined on a Gallenkamp melting point apparatus, and are uncorrected. NMR Spectra were scanned in DMSO-d_6_ on a Bruker NMR spectrophotometer operating at 500 MHz for ^1^H and 125.76 MHz for ^13^C at the Research Center, College of Pharmacy, King Saud University, Saudi Arabia. Chemical shifts are expressed in δ-values (ppm) relative to TMS as an internal standard and D_2_O was added to confirm the exchangeable protons. Mass spectra were measured on Agilent Triple Quadrupole 6410 QQQ LC/MS with ESI (Electrospray ionization) source. The X-ray diffraction measurements of compound **3c** were done using Bruker SMART APEX diffractometer. The microwave irradiations were carried out using an Explorer-48 microwave reactor (CEM, USA).

### 4.2. Conventional Synthesis of Fenamic Acid Hydrazides **3a-c**

#### 4.2.1. Step 1

A solution of the appropriate fenamic acid **1a-c** (10 mmol), absolute methanol (10 mL) and concentrated sulfuric acid (1 mL) was heated under reflux for the appropriate time (Table 1). The solvent was evaporated under reduced pressure, the remaining contents cooled to room temperature, neutralized with a concentrated solution of sodium carbonate, then the aqueous solution extracted with ether (3 times × 20 mL). The combined ether extracts were dried, and the solvent is removed under reduced pressure to yield the corresponding ester **2a-c** in 80-85% yield (Table 1).

#### 4.2.2. Step 2

A solution of hydrazine hydrate (99.9%, 5 mmol) and the appropriate methyl ester **2a-c **(1 mmol) was brought to a gentle reflux for the appropriate time (Table 1), then cooled to room temperature, The solid formed was filtered (ice/water mixture was added in some cases to complete precipitation), washed with several portions of water and dried by suction. Crystallization from EtOH afforded the corresponding fenamic acid hydrazides **3a-c** in 80-96% yield (Table 1). The reactions time of the latter two steps was 15-28 h, with overall yields of 64-86%

### 4.3. Microwave Irradiation Synthesis of Fenamic Acid Hydrazides **3a-c**

A mixture of hydrazine hydrate (99.9%, 2.5 mmol) and the appropriate fenamic acid **1a-c**(1 mmol) was irradiated, in closed vessel, under microwave irradiation at 300W and 250 °C, with 250 psi maximum pressure, for the appropriate time (Table 1). The reaction mixture was cooled; the separated solid was filtered, dried and crystallized from EtOH to give the corresponding hydrazides **3a-c **in 82-96% yields (Table 1).

#### 4.3.1. 2-(3-(Trifluoromethyl)phenylamino)benzohydrazide (**3a**)

White fibers; Mp: 136-138 °C (reported [28] 134-136 °C). ^1^H-NMR *δ*: 4.59 (br. S, D_2_O exch., 2H, NH_2_), 6.94-7.21 (m, 2H, ArH), 7.29-7.47 (m, 5H, ArH), 7.62-7.63 (m, 1H, ArH), 9.56 (s, D_2_O exch., 1H, NH), 9.91 (s, D_2_O exch., 1H, NH).^ 13^C-NMR *δ*: 113.80, 116.72, 116.91, 119.93, 120.84, 121.29, 123.01, 125.18, 128.58, 129.80-130.55 (m, -CF_3_), 131.55, 142.15, 143.10, 167.71. MS *m/z (%)*: 295 (M^+^-1).

#### 4.3.2. 2-(2,3-Dimethylphenylamino)benzohydrazide (**3b**)

White powder; Mp: 118-120 °C (reported [27] 118-120°C). ^1^H-NMR *δ*: 2.13 (s, 3H, CH_3_), 2.27 (s, 3H, CH_3_), 4.38 (br. S, D_2_O exch., 2H, NH_2_), 6.72-6.91 (m, 3H, ArH), 7.10-7.23 (m, 3H, ArH), 6.62-7.63 (m, 1H, ArH), 9.53 (s, D_2_O exch., 1H, NH), 9.88 (s, D_2_O exch., 1H, NH).^ 13^C-NMR *δ*: 13.47, 20.24, 113.87, 116.04, 116.86, 119.47, 124.97, 125.80, 128.17, 129.22, 131.73, 137.62, 139.28, 145.91, 168.45. MS *m/z (%)*: 255 (M^+^-1). 

#### 4.3.3. 2-(2,6-Dichloro-3-methylphenylamino)benzohydrazide (**3c**)

White fibers; Mp: 155-157 °C (reported [29] 158-160 °C). ^1^H-NMR *δ*: 2.38 (s, 3H, CH_3_), 4.57 (s, D_2_O exch., 2H, NH_2_), 6.21 (d, 1H, *J* = 8 Hz, ArH), 6.77 (t, 1H, *J* = 7 Hz, ArH), 7.22 (t, 1H, *J* = 7 Hz, ArH), 7.29 (d, 1H, *J* = 8 Hz, ArH), 7.48 (d, 1H, *J* = 8 Hz, ArH), 7.62 (d, 1H, *J* = 8 Hz, ArH), 9.74 (s, D_2_O exch., 1H, NH), 9.89 (s, D_2_O exch., 1H, NH). ^13^C NMR *δ*: 20.13, 113.29, 115.82, 117.62, 127.87, 128.06, 128.34, 129.27, 131.60, 132.36, 135.21, 136.39, 144.65, 168.16. MS *m/z (%)*: 310 (M^+^).
